# Maternal serum steroid hormones in vaginal delivery and caesarean section

**DOI:** 10.11613/BM.2026.010705

**Published:** 2025-12-15

**Authors:** Mirta Kadivnik, Dario Mandić, Jasenka Wagner, Kristina Kralik, Siniša Šijanović, Deni Plečko, Adrijana Muller, Gramos Begolli, Željko Debeljak

**Affiliations:** 1 Department of Obstetrics and Gynecology, University Hospital Centre Osijek, Osijek, Croatia; 2 Department of Obstetrics and Gynecology, Faculty of Medicine, J. J. Strossmayer University, Osijek, Croatia; 3 Department of Laboratory Medicine and Pharmacy, Faculty of Medicine, J. J. Strossmayer University, Osijek, Croatia; 4 Department of Medical Biology and Genetics, Faculty of Medicine, J. J. Strossmayer University, Osijek, Croatia; 5 Department of Medical Statistics and Informatics, Faculty of Medicine, J. J. Strossmayer University, Osijek, Croatia; 6 Clinic of Medical Biochemistry, University Clinical Center of Kosovo, Pristina, Kosovo; 7 Clinical Institute of Laboratory Diagnostics, University Hospital Centre Osijek, Osijek, Croatia; 8 Department of Pharmacology, Faculty of Medicine, J. J. Strossmayer University, Osijek, Croatia

**Keywords:** aldosterone, cesarean section, delivery, vaginal, steroids

## Abstract

**Introduction:**

The hormonal interplay between the mother and the fetal-placental unit may influence the mode of delivery. This study aimed to investigate the association between maternal peripartal serum concentrations of sex hormone-binding globulin (SHBG) and 10 steroid hormones with delivery outcomes.

**Materials and methods:**

This observational study included 171 healthy pregnant women with spontaneous onset of labor: 117 had vaginal delivery and 54 underwent urgent cesarean section (C-section). Serum concentrations of aldosterone, androstenedione, cortisol, cortisone, corticosterone, dehydroepiandrosterone (DHEA), dehydroepiandrosterone sulfate (DHEAS), 17-hydroxyprogesterone, progesterone, and total testosterone were determined by liquid chromatography–tandem mass spectrometry (LC-MS/MS), while free testosterone was calculated. Sex hormone-binding globulin was measured by chemiluminescent microparticle immunoassay. Group differences were tested with the Mann-Whitney U test, and associations with delivery mode were assessed by logistic regression and receiver operating characteristic (ROC) analysis.

**Results:**

Compared with the vaginal delivery group, women who underwent urgent C-section had significantly lower serum concentrations of SHBG, corticosterone, cortisol, aldosterone, progesterone, 17-hydroxyprogesterone, DHEA, DHEAS, and free testosterone (all P < 0.001). In multivariate logistic regression, aldosterone (odds ratio, OR 0.11, 95% CI 0.04 to 0.27, P < 0.001) and DHEAS (OR 0.74, 95% CI 0.58 to 0.94, P = 0.011) were independently associated with delivery mode. ROC analysis showed that aldosterone > 0.9 nmol/L predicted vaginal delivery with AUC 0.874, sensitivity 88%, and specificity 77%.

**Conclusions:**

Low maternal aldosterone concentrations showed the strongest association with urgent C-section, suggesting that aldosterone may play a protective role in successful vaginal delivery.

## Graphical abstract

**Figure:**
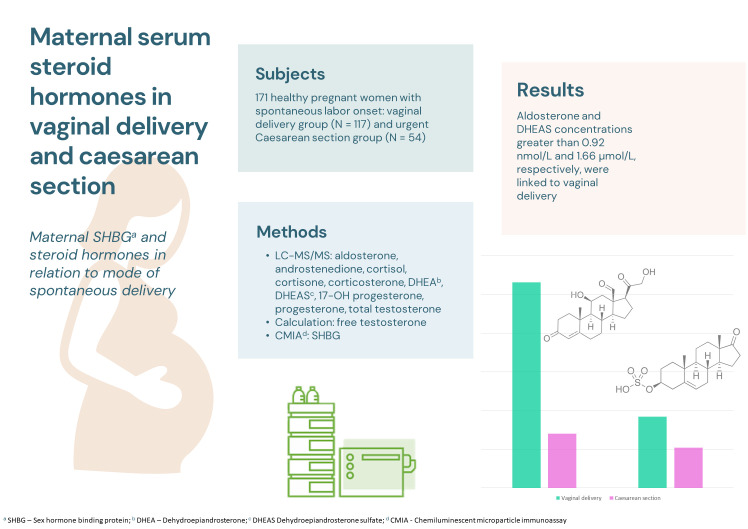

## Highlights

• The process of labor involves several key endocrine mechanisms

• Maternal serum steroids and the mode of delivery are associated

• Low aldosterone is associated with the urgent caesarean section

## Introduction

The onset of labor appears to be coordinated by complex factors related to the mother and the fetal-placental unit (*1*, *2*). It is a complex interplay of immunologic and endocrine mechanisms modulated by ethnicity, gestational age, and etiology (*3*). It includes the progressive relaxation and dilatation of the cervix, rupture of the amniotic membranes, and the initiation and maintenance of effective uterine contractions, culminating in labor (*4*). Synthesis and metabolism of steroid hormones during pregnancy are the result of complex metabolic pathways involving activities in the fetus, placenta, and mother (*5*). In humans, steroid hormones, prostaglandins, cytokines, and nitric oxide stimulate cervical remodeling by altering the content of the extracellular matrix and collagen (*6*). Cervical remodeling is of paramount importance for normal labor and it depends on dehydroepiandrosterone sulfate (DHEAS) and dihydrotestosterone (DHT) concentrations (*7*, *8*). Dehydroepiandrosterone sulfate impacts the activation of neutrophils and macrophages and the production of proteolytic enzymes in the uterine cervix needed for the vaginal delivery (*8*, *9*). Additionally, DHEAS and DHT, along with progesterone (P4) and dehydroepiandrosterone (DHEA), play a role in the contractility of the uterus (*10*). The role of maternal adrenal steroids, together with estrogen (E2) and P4, is the regulation of the fetuses’ access to nutrients, electrolytes, and water that involves control of maternal volume expansion, uterine and placental blood flow, and substrate availability (*11*). Thus, adrenal steroids may, indirectly, impact the mode of delivery.

Mode of delivery makes an important impact on the postpartum status of mothers and the perinatal outcome of newborns, especially in the active phase of delivery. Delaying an urgent Caesarean section (C-section) during the active phase of delivery, when the cervix is dilated over 6 cm and contractions are strong, can lead to significant adverse outcomes for both the mother and the fetus. The interval between the decision to perform a C-section and the actual delivery, known as the decision-to-delivery interval (DDI), is a critical factor in these outcomes (*12*). The maternal consequences of prolonged DDI could be uterine atony and postpartum hemorrhage, and a higher risk of postpartum infections, while the fetal consequences could be more frequent admission to the neonatal intensive care unit and increased perinatal mortality (*13*).

The question arises whether steroid hormones could play a predictive role in the choice of delivery mode. To our knowledge, only the study by Adamcova *et al.* has examined the relationship between the serum steroid hormone panel and mode of delivery (*5*). However, several studies investigated associations of maternal serum concentrations of individual steroid hormones, particularly androgens and cortisol with the mode of delivery (*14*-*18*). The aim of this study was to investigate the relationship between the mode of delivery and peripartal, maternal serum concentrations of an extended steroid panel consisting of aldosterone, androstenedione, cortisol, cortisone, corticosterone, DHEA, DHEAS, 17-hydroxyprogesterone (17-OHP), P4, free and total testosterone, along with the sex hormone-binding globulin (SHBG).

## Materials and methods

### Subjects

This observational study was conducted between November 2017 and April 2023 at the Department of Gynecology and Obstetrics and the Clinical Institute for Laboratory Diagnostics, Osijek University Hospital. Total of 171 healthy pregnant women whose delivery began with a regular spontaneous contraction of the uterus with or without premature membrane rupture, were included in the study. Inclusion criteria for our study involved a singleton pregnancy with a newborn in cephalic presentation: mothers were admitted in the first stage of delivery, and had regular contractions. Exclusion criteria were the use of drugs that influence steroidogenesis, hyper- or hypothyroidism, pregnancy complications (hyperemesis, bleeding, endometriosis, any previous operation on the uterus), and multiple pregnancies. Subjects were divided into 2 groups: the first group was composed of 54 woman whose labor started spontaneously, but ended with the urgent C-section due to indications that were not predictable before the onset of labor (*e.g*., cervical-corporal dystocia, fetal hypoxia, vaginal hemorrhage), while the second group was composed of 117 woman whose labor started spontaneously and ended with vaginal delivery. This study has been a part of a larger project: although it shares participants with the previous study, the study goals are different (*19*). Demographic data included the mothers’ age, body mass index (BMI), parity, and smoking status, together with the newborns’ gender and weight. Body mass index was categorized as normal (18.5-24.9), overweight (25-29.9), and obese (≥ 30) according to WHO criteria (*20*). Macrosomal newborns are defined as a birth weight of newborn relative to gestational age, specifically at or above the 90th percentile (*21*). The questionnaire collected information on maternal age, maternal medical history, maternal smoking status, newborns gender, and newborns weight. Demographics are given in Table 1.

**Table 1:** Demographic characteristics of women undergoing urgent cesarean section and those with vaginal delivery

	**Urgent cesarean section** **(N = 54)**	**Vaginal delivery** **(N = 117)**	**P value**
Mothers’ age (years) [Median (min - max)]	31 (20-41)	30 (18-44)	0.581^†^
BMI (kg/m^2^) [Median (IQR)]	26.8 (23.6-30.5)	26.4 (24.2-29.4)	0.673^†^
BMI [N (%)]			
Normal weight	17 (31)	42 (36)	
Overweight	22 (41)	47 (40)	0.807*
Obese	15 (28)	28 (24)	
Parity [N (%)]			
Primiparous	40 (74)	49 (42)	< 0.001
Multiparous	14 (26)	68 (58)	
Mothers smoking habit [N (%)]	18 (33)	32 (27,4)	0.42
RVP [N (%)]	17 (31)	38 (32,5)	0.89 *
Weeks of gestation [Median (IQR)]	38+6 (35+6 – 39 + 3)	38+5 (36+2 – 39+4)	0.944^†^
Newborns gender [N (%)]			
Male	29 (54)	62 (53)	0.931*
Female	25 (46)	55 (47)	
Newborns birth weight (g) Median (IQR)	2999 (2151 - 3300)	3280 (2795 - 3593)	**0.009**
Macrosomal newborns			
No	54 (100)	107 (91.5)	**0.03**
Yes	0	10 (8.5)	
Bold denotes statistically significant differences. *χ^2^ test; ^†^Mann-Whitney U test. Percentages within each group, column totals sum to 100%. Macrosomal newborns are defined as a birth weight of newborn relative to gestational age, specifically at or above the 90th percentile (21). Gestational age is expressed as completed weeks of gestation + days (*e.g.,* 38+6 = 38 weeks and 6 days). Values are presented as median (interquartile range). N - number of participants. BMI - body mass index; BMI categories defined according to WHO criteria (20). RVP – lat. *ruptura velamentorum praetemporaria*.			

This study has been conducted in accordance with the World Medical Association Declaration of Helsinki (*22*). It was approved by the Ethical Committee of the University Hospital Osijek (approval number: R2: 12272-4/2017) and the Faculty of Medicine, Josip Juraj Strossmayer University of Osijek (approval number: class: 602-04/18-08/07; Reg. No: 2158-61-07-18-133). All participants signed an informed consent form and filled out a questionnaire requiring relevant medical history and socio-demograhic information. Participants´ anonymity was guaranteed.

### Blood sampling and analysis

After the patients were admitted to the delivery room, venous blood was drawn in test tubes that did not contain anticoagulants. Blood samples were obtained during the first stage of labor, before the application of the oxytocin infusion, if needed, and predominantly in the supine position; however, variations in maternal posture could not be fully controlled and may have influenced aldosterone concentrations. Venous blood (5 mL) was collected in plain serum separator tubes (BD Vacutainer, Becton Dickinson, Franklin Lakes, USA) during the first stage of labor, before oxytocin infusion, if applicable. Samples were centrifuged at 3500 rpm for 10 minutes, aliquoted, and stored at - 70 °C until analysis.

A liquid chromatography-tandem mass spectrometry (LC-MS/MS) instrument, LCMS-8050 (Shimadzu, Kyoto, Japan) was used to determine of the serum concentration of 10 steroid hormones (aldosterone, androstenedione, cortisol, cortisone, corticosterone, DHEA, DHEAS, 17-OHP, P4 and testosterone). All serum samples for LC-MS/MS determination of steroid hormones were prepared using the same standardized procedure (ChromSystems kit), while SHBG concentration was determined separately by chemiluminescent immunoassay. The determination of hormone serum concentrations was preceded by a serum sample preparation using a commercially available kit (ChromSystems, Gräfelfing, Germany). Equation by Vermeulen *et al.* was used to calculate the serum concentration of free testosterone. The concentration of SHBG was measured using Abbott ARCHITECT i1000SR chemiluminescent microparticle immunoassay (Abbott Diagnostics, Lake Forest, USA).

### Statistical analysis

Categorical variables were presented as counts and percentages, and compared using the chi-square test. Normality of continuous variables was assessed with the Shapiro–Wilk test. Continuous variables were summarized as medians with interquartile range or range (minimum-maximum) and compared between groups using the Mann–Whitney U test. In the bivariate logistic regression analysis, the following variables were considered as independent predictors: SHBG, corticosterone, cortisol, cortisone, aldosterone, progesterone (P4), 17-hydroxyprogesterone (17-OHP), DHEA, DHEAS, androstenedione, testosterone, and free testosterone. Associations between these variables and mode of delivery were examined with bivariate and multivariate logistic regression. Multivariate models were fitted using the stepwise method (entry P < 0.05, removal P > 0.2). Results were expressed as odds ratios (OR) with 95% confidence intervals. The goodness-of-fit of the model was assessed using the Hosmer–Lemeshow test. Receiver operating characteristic (ROC) curve analysis was applied to evaluate the diagnostic performance of significant predictors. All P values were two-sided, with the level of significance set at α = 0.05. Analyses were performed using SPSS 17.0 (SPSS Inc., Chicago, USA) and MedCalc 23.3.7 (MedCalc Software Ltd, Ostend, Belgium).

## Results

The baseline characteristics of all study participants and the difference in sociodemographic characteristics between the two groups of patients who had vaginal deliveries or C-sections are shown in Table 1.

**Figure 1:**
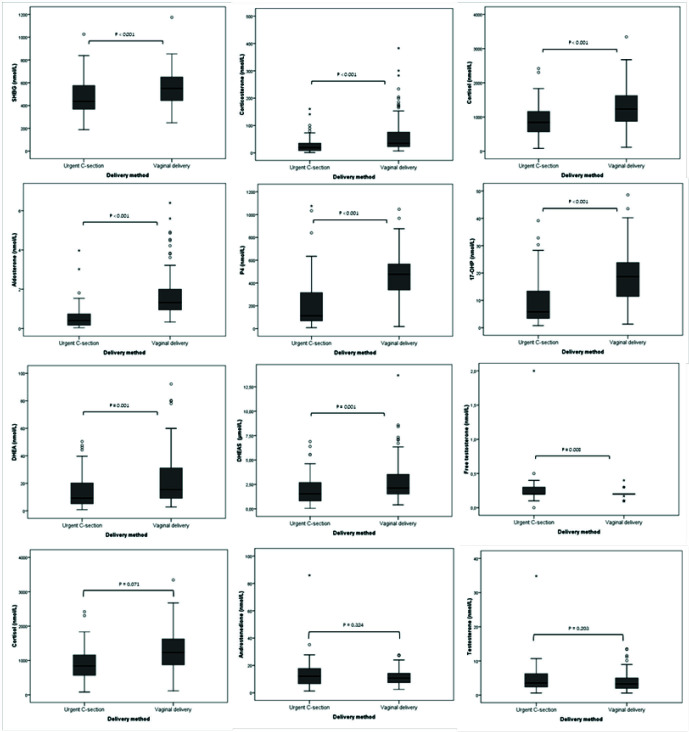
Box and whisker plot showing the differences in hormone concentrations between urgent C-section and vaginal delivery. The Mann-Whitney U test was used. SGBG - Sex Hormone Binding Globulin. P4 - progesterone. 17-OHP - 17-hydroxyprogesterone. DHEA - dehydroepiandrosterone. DHEAS - dehydroepiandrosterone sulfate. C-section - Caesarean section.

A statistically significant difference between groups was found in serum concentrations of SHBG, corticosterone, cortisol, aldosterone, P4, 17-OHP, DHEA, DHEAS, and free testosterone (Figure 1). All of these serum concentrations were lower in the group of patients whose labor ended with an urgent cesarean section. Similar results were obtained by bivariate logistic regression, and the most important results were as follows: lower aldosterone (OR 0.09, 95% CI 0.04 to 0.23, P < 0.001) and lower DHEAS concentrations (OR 0.74, 95% CI 0.59 to 0.93, P = 0.009) were significantly associated with urgent C-section. Testosterone and cortisone, and free testosterone, did not demonstrate statistically significant differences between groups, while other steroid hormones and SHBG showed a modest association with urgent C-section. In the multivariate model adjusted for parity and fetal macrosomia, aldosterone, DHEAS, and SHBG remained independent predictors of urgent C-section. They were inversely associated with the C-section as a mode of delivery. Multivariate regression analysis (Table 2) was used to develop a fully statistically significant model (χ2 = 70.3, P = 0.001). The model accurately classified 85% of cases. Regression analysis was performed with correction for confounding factors (maternal parity and fetal macrosomy). Aldosterone and DHEAS have significantly contributed to the above model, demonstrating a protective role: the higher the serum concentration of aldosterone and DHEAS, the lower the likelihood of C-section (OR 0.11 and OR 0.74, respectively).

**Table 2:** Multivariate logistic regression analyses predicting the probability of cesarean delivery

**Variables**	*Multivariate regression analysis			
	β	P value	OR	95% CI
SHBG	- 0.322	**0.004**	0.99	0.99 to 0.99
Aldosterone	- 2.096	**< 0.001**	0.11	0.04 to 0.27
DHEAS	- 0.322	**0.020**	0.74	0.58 to 0.94
Constant	4.787	**< 0.001**		
*adjusted for fetal macrosomia and parity. Bold font denotes statistically significant differences. SGBG - sex hormone binding globulin. DHEAS - dehysdroepiandrostendion sulfate. OR - odds ratio. 95% CI - 95% confidence interval. β - regression coefficient.				

**Figure 2:**
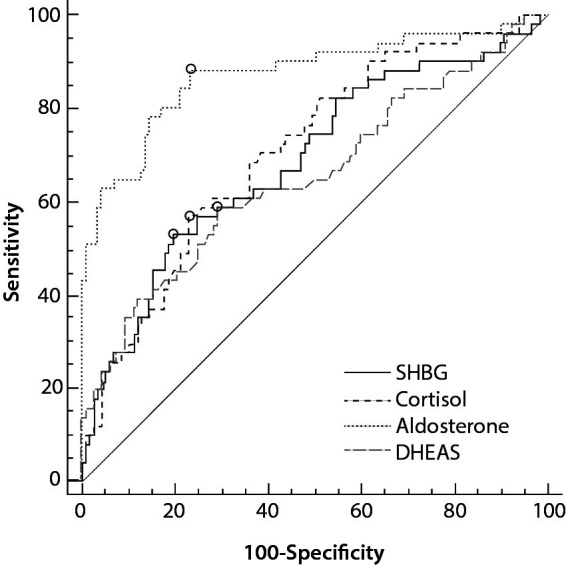
Receiver operating characteristic (ROC) curves of maternal SHBG, cortisol, aldosterone, and DHEAS illustrating their association with mode of delivery (caesarean section *vs.* vaginal delivery). Aldosterone demonstrated the highest discriminatory accuracy among the analyzed hormones. SHBG - sex hormone binding globulin. DHEAS - dehydroepiandrosterone sulfate.

Based on the ROC analysis, aldosterone proved to be the most important indicator of urgent C-section (Table 3 and Figure 2). Aldosterone concentration greater than 0.9 nmol/L was associated with vaginal delivery (area under the curve (AUC) = 0.874, sensitivity = 88%, specificity = 77%, P = 0.001), while the rest of the investigated steroids provided less pronounced differences between delivery modes.

**Table 3:** Receiver operating characteristic (ROC) analysis of maternal hormone parameters in relation to mode of delivery (caesarean section *vs* vaginal delivery)

**Parameters**	**AUC**	**95% CI**	**Sensitivity%**	**Specificity%**	Cut-off*	**Youden**	**P value**
SHBG	0.673	0.598 to 0.743	50	80	≤ 431.1	0.303	**< 0.001**
Cortisol	0.709	0.635 to 0.776	69	64	≤ 1093	0.326	**< 0.001**
Aldosterone	0.874	0.814 to 0.920	88	77	≤ 0.9	0.652	**< 0.001**
DHEAS	0.653	0.576 to 0.724	57	71	≤ 1.7	0.284	**0.001**
Bold font denotes statistical significance. All concentrations are given as nmol/L. SHBG - sex hormone binding globulin. DHEAS - dehysdroepiandrostendion sulfate. AUC - area under the curve. 95% CI - 95% confidence interval.							

Additional analyses showed that newborns delivered by urgent C-section had significantly lower birth weight compared with those born vaginally (Table 1). Macrosomia occurred only in the vaginal delivery group (Table 1, 8.5% *vs.* 0%, Fisher’s exact test P = 0.03). In parity-stratified analyses, multiparous women had significantly higher concentrations of SHBG (OR 1.52, 95% CI 1.12 to 2.07, P = 0.007) and aldosterone (OR 1.38, 95% CI 1.07 to 1.79, P = 0.01), whereas primiparous women had higher androstenedione (OR 1.71, 95% CI 1.30 to 2.25, P < 0.001) and testosterone concentrations (OR 1.95, 95% CI 1.43 to 2.66, P < 0.001). No significant hormonal differences were observed according to the presence or absence of prelabor rupture of membranes (PROM).

## Discussion

During pregnancy, P4, E2, androgens, and corticosteroids play an important role, and their concentrations vary depending on the type of delivery (*5*). In a spontaneous vaginal delivery, however, the concentrations change due to the onset of labor and the stress response to delivery (*5*). To our knowledge, no studies that analyze the possible link between serum concentrations of steroid hormones determined by LC-MS/MS and urgent C-section have been published. To bridge this gap, in the present study, the focus was on differences between serum concentrations of SHBG and 10 selected maternal steroid hormones between two groups of mothers divided by the mode of delivery and their connection with the mode of delivery. It was found that there was a significantly lower concentration of SHBG, corticosterone, cortisol, aldosterone, P4, 17-OHP, free testosterone, DHEA, and DHEAS in the group of patients whose birth ended with an urgent C-section. A similar study performed by Adamcova *et al.* also detected differences in maternal serum steroids between the modes of delivery (*5*). Still, in comparison to our study, aldosterone was not included in the study, and these authors compared serum concentrations of steroid hormones between groups of patients whose labor ended by vaginal delivery or by the elective C-section. There is an expected difference in the serum concentration of steroid hormones between urgent and elective C-sections: in urgent C-sections, there is a possibility that certain steroid serum concentrations are more consistent with serum concentrations of steroids in vaginal delivery.

The link between maternal serum androgens and the mode of delivery was also obtained by other groups (*14*-*18*). The only exception from the established link is represented in the study by Cawyer *et al*., these authors were unable to establish a clear correspondence between DHEAS concentrations and the mode of delivery, which may be attributed to the different analytical method used in this study (*23*).

Particularly interesting results from the study presented here came from the bivariate and multivariate logistic regression: aldosterone, but also DHEAS, provide a statistically significant contribution to regression models of a delivery mode. The ROC analysis revealed a cut-off value below which the C-section is expected. Among the given hormones, aldosterone, with an OR of 0.107 for the urgent C-section, stands out. Adrenal steroids may influence the type of birth *via* indirect control. For example, in response to the stress caused by labor and vaginal delivery, aldosterone concentration rises during vaginal delivery more than during urgent C-section (*24*). Jensen *et al.* demonstrated that adrenal steroid hormones, together with E2 and P4, regulate fetal access to nutrients, electrolytes, and water by regulating maternal volume expansion, uterine and placental perfusion (*11*). Aldosterone itself affects placental blood flow and development, as well as the transfer of oxygen and nutrients to the fetus (*25*-*27*). Uterine contractions could favor the occurrence of fetal asphyxia and/or hypoxia and termination of pregnancy by C-section, if angiogenesis and placental development are inadequate, which may be associated to the aldosterone concentration below 0.9 nmol/L. A significant influence of aldosterone on the course of labor could also lie in its influence on the expansion of plasma volume.

As presented in the Results section, we found significantly lower concentrations of SHBG, corticosterone, cortisol, aldosterone, progesterone, 17-OHP, free testosterone, DHEA, and DHEAS in women whose delivery ended with urgent C-section. These results suggest relative hypovolemia in pregnant women who underwent C-section, which may be a consequence of a lower serum concentration of aldosterone in the C-section group. Insufficient aldosterone production may lead to inadequate expansion of plasma volume, which may result in uteroplacental insufficiency. This condition can impair uterine contractility and cervical ripening and lead to dysfunctional labor patterns such as prolonged or stopped labor. As a result, the likelihood of interventions such as induction of labor or C-section increases (*28*). All presented results and explanations suggest a possible protective role of maternal serum aldosterone against the urgent C-section.

Possible influence of androgens, especially DHEAS, on the mode of labor can be explained by their effect on cervical remodeling and myometrial contractility (*9*, *27*). The effect of DHEAS on cervical remodeling has been associated with increased levels of the cytokine IL-8, which is involved in the chemotaxis of neutrophils recruited to the cervix towards the end of pregnancy, and it stimulates the secretion of proteolytic enzymes (*9*, *28*). In short, lower DHEAS concentrations (< 1.7 nmol/L) could lead to a lack of peripartum cervical remodeling and thus urgent C-section. Inversely, the increase of the DHEAS concentration lowers the risk of C-section (OR 0.76), suggesting a possible protective role of DHEAS against the urgent C-section. Remarkably, only 2 steroids in the group that underwent C-section had higher serum concentrations, namely testosterone and androstenedione. It is important to mention that these differences were not statistically significant. It could be due to a difference in renal function between the two groups of pregnant women, and this could be the basis for some future study. Although renal clearance differences could be one possible explanation, as suggested by recent evidence linking kidney function and female reproductive hormones, this parameter was not assessed in our study and should therefore be interpreted with caution (*29*).

In our cohort, primiparity was significantly more frequent in the urgent C-section group, which is consistent with its established role as a risk factor for cesarean delivery. To account for this potential bias, parity (together with fetal macrosomia) was included as a covariate in the multivariate regression analysis. Importantly, aldosterone, DHEAS, and SHBG remained independent predictors of urgent C-section even after adjustment, indicating that the observed associations were not explained by differences in parity.

Our finding of no significant differences in cortisol (or other steroid hormones) between women with and without rupture of membranes is noteworthy. Although cortisol has been shown to dramatically induce matrix metalloproteinase-7 (MMP-7) in the human amniotic tissue, leading to degradation of the extracellular matrix, and elevated MMP-9 expression has been associated with spontaneous rupture of fetal membranes, our data do not support a role for measurable systemic steroid hormone differences by RVP status in our cohort (*30*, *31*). Local (membrane-level) expression/activity of MMPs may be a more sensitive indicator than circulating hormone concentrations.

There are some specific limitations and some strengths of the presented study. The timing of sampling could be seen as such limitation. Sampling took place at different stages of the first stage of delivery, depending on when the subjects were admitted to the delivery room. In addition, a C-section was performed shortly before the delivery, when the labor-induced hormonal changes had already started. Future research should be conducted over several weeks before delivery as a longitudinal study of hormonal changes. In addition, the limitations of this study include a relatively small number of women who underwent urgent C-section and the lack of follow-up measurements before delivery. Furthermore, another limitation of our study is the lack of strict control for maternal posture at the time of blood sampling. Postural changes are known to influence aldosterone concentrations, and although samples were generally collected in the supine position, variability cannot be fully excluded (*32*). At the end, one of the limitations could be the fact that some predictors in bivariate regression yielded narrow 95% confidence intervals, which may be related to the relatively low variability of the measurements. To improve interpretation, the results should be considered in the context of clinically relevant units of change.

The main strength of this study was the analytical method by which serum concentrations of steroids were determined. Measurements of the serum steroids by immunoassays were found to show a significant analytical bias (*28*). Due to chromatographic separation and the selectivity of mass spectrometry, LC-MS/MS is less susceptible to interference from related compounds that may be present in maternal serum (*19*, *28*). No studies dealing with the association of free testosterone with the mode of delivery were found.

In conclusion, maternal serum concentrations of DHEAS and aldosterone are associated with mode of delivery. Low aldosterone concentrations were linked to an increased likelihood of urgent cesarean section, suggesting a possible protective role of aldosterone in successful vaginal delivery. Further multicentric studies are needed to confirm this role.

## Data Availability

Data presented in this study is available on request from the corresponding author.
